# Determinants of pelvic organ prolapse at public hospitals in Hawassa city, Southern Ethiopia, 2020: unmatched case control study

**DOI:** 10.1186/s12905-022-01890-z

**Published:** 2022-07-20

**Authors:** Bezabih Terefe Dora, Zemenu Yohannes Kassa, Nebiha Hadra, Bamlaku Birie Tsigie, Hawi Leul Esayas

**Affiliations:** 1 Department of Midwifery, College of Medicine and Health Sciences, Arbaminch University, Arbaminch, Ethiopia; 2 Department of Midwifery, College of Medicine and Health Sciences, Hawassa University, Hawassa, Ethiopia; 3 Department of Midwifery, College of Medicine and Health Sciences, Mizan Tepi University, Mizan, Ethiopia

**Keywords:** Pelvic organ prolapses, Hospitals, Determinants, Hawassa city

## Abstract

**Introduction:**

Even though the Pelvic organ prolapse (POP) is outstanding gynecologic problem, most private and asymptomatic nature of the illness makes it the “hidden epidemic.” The aim of this study was to identify the determinants of POP.

**Methods:**

Facility based unmatched case control study was conducted from June 15 to September 10, 2020. All cases diagnosed with POP were enrolled in the study by using consecutive random sampling method by assuming that patient flow by itself is random until the required sample size was obtained. Then 1:2 cases to control ratio was applied. A structured interviewer-administered questionnaire and chart review for type and degree of prolapse was used. Epi-data was used for data entry and SPSS were used for analysis. Chi square test and binary and multivariable logistic regression analysis was employed. Multicollinearity was checked.

**Result:**

On multivariate logistic regression, heavy usual work load(AOR = 2.3, CI(1.066–4.951), number of pregnancy ≥ 5(AOR = 3.911, CI(1.108–13.802), birth space of < 2 years(AOR = 2.88, CI(1.146–7.232), history of fundal pressure (AOR = 5.312, CI(2.366–11.927) and history of induced labor (AOR = 4.436, CI(2.07–9.505) were significantly associated with POP with *P* value < 0.05 and 95% CI after adjusting for potential confounders.

**Conclusion:**

Heavy usual work load, having pregnancy greater than five, short birth space, history of induced labor, and history of fundal pressure are independent predictors of pelvic organ prolapse. Hence the responsible body and obstetric care providers should counsel the women about child spacing, minimizing heavy usual work load and effect of multigravidity on POP. Incorporation of health education on those risk factors related to POP on antenatal and postnatal care should be considered. The obstetric care providers also avoid fundal pressure and labor induction without clear indication and favorability, and the hospital officials set a law to ban fundal pressure during labor.

## Introduction

Pelvic organ prolapse (POP) is a gynecologic morbidity in which one or more of the female pelvic organs, such as the bladder, uterus, vaginal cuff, rectum and intestine, descend through the vagina [1, 2]. It is a result of anatomic support defect of the pelvic viscera caused by combined series of long term failure of pelvic floor muscle, connective tissue and pubococcygeal portion of the levator ani muscle which leads to defect of supporting and suspension mechanisms of the uterus and the vaginal wall ending in downward displacement of structures that are normally located adjacent to the vaginal vault[3, 4]. POP has been a major health concern throughout the world affecting millions of reproductive and menopause aged women with estimated lifetime prevalence of 30.0% to 50.0% [5], commonly affecting elderly and multiparous women than those under the age of 45 years (2–20% and 39.8%) covering estimated global prevalence of 2.9%–20% [6]

Due to the harsh environment related to socioeconomic, geographic and institutional factors, the burden of POP has been significant in developing countries than developed countries. The incidence of POP in low and middle income countries was 19.7% with the range of 3.4%–56.4% [7]. In Nepal, more than 600,000 women suffer from POP and 200,000 women require pelvic surgery for genital prolapse [5]. About (12%) in Ghana, 46% in Gambia and 37.5 in Nigeria per 1000 gynecological admission were suffered from POP [6, 8, 9]

The burden of POP in Ethiopia is high with estimated prevalence of 23.52% [10]. The burden of high fertility, early pregnancy rate and limited access to obstetric care and health services and the condition’s strong association with shame and stigma in the country [7] is a reason to believe that pelvic organ prolapse constitutes a major women’s health challenge in Ethiopia [11]. It accounted for 40.7% of major gynecological operations in Jima and (41.1%) of hysterectomy followed by leiomyoma (23%) in Tikur Ambesa [12]. Study done in Tigray region also indicated that 12 out of 1000 women were suffered from POP [6]

The determinants of POP may be grouped into several categories, including intrinsic (genetics, age, postmenopausal status, ethnicity) and extrinsic factors (obstetric history, co-morbidities, occupation).

POP can severely affect a woman’s quality of life with a great negative impact on women’s social, physical, and psychological wellbeing. In addition, POP and its complications impose a considerable economic burden due to reduced quality of life, impairment of workforce productivity, and cost to both the individual and the health care system as a whole. Depression and disrupted marital relationship followed by divorce were also another series impact of POP [6, 8, 13–17].

Even though the problem is outstanding, most secretive and asymptomatic nature of the illness makes POP the “concealed sweeping” and the true incidence and determinant factors have been not sufficiently identified. Although few studies concerned with determinant factors of POP have been documented they were not nationally representative and lack appropriate design and power of analysis and all suspected factors were not considered. Besides, due to complex and multi-factorial nature of its etiology and the determinant differ geographically from patient to patient; authors on their study and ministry of health recommends further study in different area, but to the extent of my searching, there is no representative study with similar design was conducted concerning the topic in my study area.

Despite of its burden, there is no POP targeted initiatives and interventions have been done at the country level rather than campaign treatment of already diagnosed POP cases and strengthening family planning service targeting on birth spacing. Also, there is no independent guide on preventive measures and health promotion activity targeted on POP. So, the availability of local information on the determinant factors of POP has a major role in the management and control of the case in that area and gaining insights of such factors from this study will help the stake holders and implementers to design new as well as to strength the existing programs towards the problem. Hence, the objective of this study was to identify the determinants factors of POP in Hawassa city public hospitals, Southern Ethiopia, 2020.

## Methods and materials

### Study setting

The study was conducted at public hospitals in the Hawassa city. Hawassa is an administrative city of SNNPRS and Sidamo regional state found in about 275 km to south of Addis Ababa. It is in the great rift valley of central Ethiopia and lies at the eastern edge of large Lake Hawassa, with its resident hippos. Hawassa city found in elevation of 1708 m and weather condition of 18 °C, wind NE at 2 km/h, and humidity of 80%. Amora Gedel National Park, Lake Hawassa and St. Gebriel church were known tourist centers for visitors. According to the report of city health office, the city has total number of populations 385,237 from these 191,858 were women and 193,399 were men. The number of reproductive groups was 89,765. There are three government hospitals named Hawassa comprehensive specialized teaching and referral, Adare general and Tula primary hospital, and five private hospitals serving for about 5.53 million populations including attendants from in and around SNNPRS as their catchment population. There are about 6792 women who visit the three government hospitals for gynecologic service each year.

### Study design and population

This was facility based unmatched case control study conducted to identify the determinants of Pelvic organ prolapse. The populations were all women who visit gynecologic unit at public hospitals in Hawassa city public hospital from June 15 to September 10, 2020. A total of 231 (77cases and 154 controls, with case to control ratio of 1: 2.) sample size by including 10% non-response rate were included in the study.

### Eligibility criteria

Included cases were women’s with confirmed cases of pelvic organ prolapse in gynecologic clinic excluding first degree of UVP by physician at Hawassa city public Hospitals. Included controls were a woman’s who came for other gynecologic problem rather than pelvic organ prolapse during the same period as of the cases. A severely ill, previously hystrectomized women’s and clients diagnosed with first degree UVP (stage I) were excluded.

### Study variables

Dependent variable was pelvic organ prolapses and independent variables includes Socio demographic characteristics (Age, educational status, occupation (type), work load, place of residence, house hold monthly income, medical and personal conditions (Smoking, alcohol taking, constipation, chronic cough, Diarrhea, DM, Current BMI, Family history of POP, information about POP) and obstetric condition (Gravidity, Parity, age at first birth and marriage, mode of delivery, menstrual status, type of delivery, place of delivery, type of birth attendant(TBA or SBA), instrumental delivery, episiotomy, induced labor, fundal pressure, FP utilization, ANC, history of abortion, perineal damage/tear in previous birth, birth spacing, duration of labor, rest after birth in puerperium, birth weight).

### Operational definition

#### Heavy work load

Those a usual task involving lifting of heavy object/ doing extensive physical labor that strains the pelvic organs such as farming, looking for cows, sheep, and goat herd, carrying and marketing of agricultural products, wood collection, fetching water and preparing kocho /false banana [4, 18]

#### Light workload

Includes all tasks that do not involve a usual lifting of heavy objects/works didn’t requires heavy force to strain the pelvic organ [13, 18]

#### Chronic cough

Having cough for fourteen days duration resulting in high intra-abdominal pressure [8]

#### Chronic constipation

Having difficulty of passing feces which result in high straining of abdominal and pelvic muscles which occurred one or more times per month [13]

#### Physical violence on reproductive organs

Having the history of blunt kick or sharp injury to reproductive organs and perineum either from intimate partner or others.

#### History of smoking

Having history of smoking in the last 12 months.

#### Alcohol taking

Having the history of 2 drinks or more (more than moderation) in a day (CDC Dietary Guidelines for Alcohol 2020–2025).

### Data collection procedure and tool

For three (3) hospitals in the city proportional size allocation was done based on their case flow to reach the minimum required sample size. All cases diagnosed with POP except first degree UVP were enrolled in the study by using consecutive random sampling method by assuming that patient flow by itself is random until the required sample size was obtained. Data were collected after the physician (health team/specialist) confirmed the stage and type of POP by using standard diagnostic methods. Then for each eligible case, two controls were selected consecutively at the same time immediately following the identified cases. The structured interviewer administered questionnaire developed from existing literature and chart review was used for data collection. By trained midwifes, detailed socio-demographic, obstetric, medical, and personal related history was taken by using the Amharic (local language) version questionnaire.

### Data quality management and analysis

The questionnaire was prepared in English and translated to Amharic & then back to English prior to the start of field work to make sure that the questions were clear, understood by the respondents and kept consistence. Recruited data collectors and supervisors were trained on purpose of the study, selection of cases exposed and unexposed, getting consent from the woman, how to keep confidentiality of information, the contents of the questionnaire and data quality management by the investigators based on the guide that was developed for clarifying interview questionnaires.

Under close daily supervision, the collected data were checked for completeness and consistency by the investigator too and then cleaned and entered into EPI- data version 3.1 and then exported in to SPSS Version 20 for analysis. Descriptive statistics was computed and described using tables, figures, and texts. Chi-square test was done initially before binary logistic regression to check whether there is base line difference or not in two groups. Then binary and multivariable logistic regression model was used to identify the association between explanatory and outcome variables. Variables with *p*-value < 0.2 in binary logistic regression were candidate for multivariable logistic regression and the fitness of model was checked with Hosmer and Lemshew test and multicollinearity was checked. OR with 95% CI was calculated to measure the strength of association between explanatory variables and the outcome variable. Finally, *p*-value < 0.05 were considered as statistically significant.

## Results

### Sociodemographic characteristics

A total of 231 study participants (77 cases with pelvic organ prolapse and 154 controls without pelvic organ prolapse) were participated, making the response rate of 100%. About 138(59.7%) participants were in the age group of ≥ 40 years, with a mean and standard deviation of age of 45.26 ± 14.77 years. Compared to the control groups, about three-fourth of cases were in the age group of ≥ 40 (60(77.9%)) (Table 1).

**Table 1:** Sociodemographic characteristics and information about POP of respondents in Hawassa city public hospitals, 2020

Variables	Controls(154)	Cases(77)	X^2^	*P*
Age grouped				
< 40	76(49.4%)	17(22.1%)		
> = 40	78(50.6%)	60(77.9%)	15.9	0.000
Residence of the respondent’s				
Urban	101(65.6%)	34(44.2%)		
Rural	53(34.4%)	43(55.8%)	9.7	0.002
Religion of the respondent’s				
Orthodox	35(22.7%)	21(27.3%)		
Islam	32(20.8%)	18(23.4%)		
Protestant	71(46.1%)	27(35.1%)		
Catholic	11(7.1%)	8(10.4%)		
Other	5(3.2%)	3(3.9%)	2.8	0.593
Job types of the respondent’s				
House wife	62(40.3%)	33(42.9%)		
Merchant	32(20.8%)	13(16.9%)		
Government employee	22(14.3%)	15(19.5%)		
NGO employee	13(8.4%)	8(10.4%)		
Daily labor	14(9.1%)	8(10.4%)		
Student	11(7.1%)	0(0.0%)	7.15	0.209
Work load of the respondent’s				
Light work	99(64.3%)	25(32.5%)		
Heavy forceful work	55(35.7%)	52(67.5%)	20.9	0.000
Marital status of participants				
Married	16(82%)	48(62.3%)		
Divorced	21(13.6%)	11(14.3%)		
Widowed	28(18.2%)	18(23.4%)	0.98	0.614
Educational label				
Illiterate	50(32.5%)	30(39.0%)		
Read and write	17(11.0%)	2(2.6%)		
Primary and secondary	61(39.6%)	41(53.2%)		
Higher education	26(16.9%)	4(5.2%)	12.6	0.005
Household monthly income				
< = 1025	85(55.2%)	51(66.2%)		
1026–3995	26(16.98%	19(24.7%)		
> 3995	43(27.9%	7(9.1%)	11.07	0.004

### Personal, family and medical related characteristics of the respondents

More than half (139(60.2%) of respondents had no information about pelvic organ prolapse from this the case group covers more (74.0%) (Table 2).

**Table 2:** Personal, family and medical related characteristics of the respondents in Hawassa city public hospitals, 2020

Variables	Controls	Cases(77)	X^2^	*P*
The family history of respondent’s with POP				
Yes	15(9.7%)	15(19.5%)		
No	139(90.3%)	62(80.5%)	4.3	0.038
The respondents history of chronic cough				
Yes	41(26.6%)	33(42.9%)		
No	113(73.4%)	44(57.1%)	6.2	0.013
The respondents history of constipation				
Yes	39(25.3%)	34(44.2%)		
No	115(74.7%)	43(55.8%)	8.422	0.004
Respondents history of diarrhea				
Yes	20(13.0%)	8(10.4%)		
No	134(87.0%)	69(89.6%)	0.325	0.569
BMI grouped				
18.5–24.9	127(82.5%)	63(81.8%)		
< 18.5	19(12.3%)	12(15.6%)		
> = 25	8(5.2%)	2(2.6%)	1.2	0.547
Respondent’s history of DM				
Yes	14(9.1%)	8(10.4%)		
No	140(90.9%)	69(89.6%)	0.1	0.751
Respondents history of smoking				
Yes	10(6.5%)	8(10.4%)		
No	144(93.5%)	69(89.6%)	1.085	0.298
Alcohol taking				
Yes	40(26.0%)	19(24.7%)		
No	114(74.0%)	58(75.3%)	0.046	0.831
Respondent’s history of STI				
Yes	39(25.3%)	24(31.2%)		
No	115(74.7%)	53(68.8%)	0.885	0.347
Information about POP				
Yes	72(46.8%)	20(26.0%)		
No	82(53.2%)	57(74.0%)	9.25	0.002
Information about aggravating factor of POP				
Yes	72(46.8%)	22(28.6%)		
No	82(53.2%)	55(71.4%)	7.032	0.008
Information about the cause of POP				
Yes	72(46.8%)	31(40.3%)		
No	82(53.2%)	46(59.7%)	0.876	0.349

### Obstetric and gynecologic related characteristics of cases and controls

**Fig. 1:**
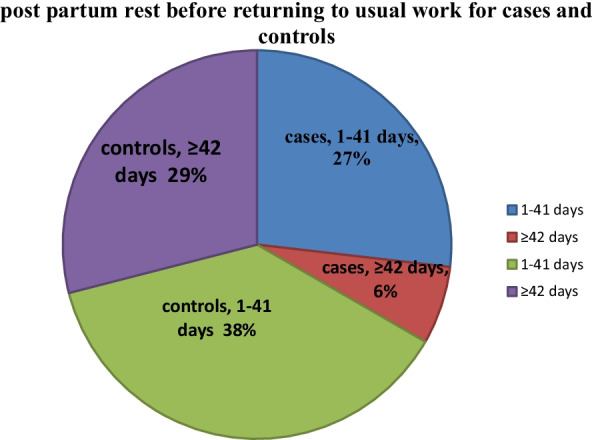
Respondent’s post-partum rest before returning to usual work at Public Hospitals in Hawassa City, Southern Ethiopia,

**Fig. 2:**
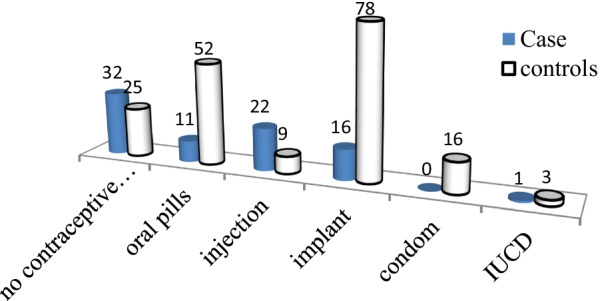
Modern contraceptive use of the respondents at Public Hospitals in Hawassa City, Southern Ethiopia, 2020

Sixty-seven (87%) of the cases had more than five childbirth, whereas only 54.5% of controls had ≥ 5 childbirth. Compared to control groups, more than three-fourth of cases gave birth via spontaneous vaginal childbirth (60(77.9%). (Table 3.) From total participants, about seventy five percent (149(64.5%)) return to their usual work before 42 days of post-partum period and compared to the control groups, most of case group return to their usual work before 42 days (80.5%)(Fig. 1). Compared to controls, more than half of cases were none users of modern contraceptive methods and high utilization of short acting than long acting (Fig. 2).

**Table 3:** Obstetric and gynaecologic related characteristics of cases and controls Hawassa city public hospitals, 2020

Variables	Category	Controls n = 154	Cases n = 77	X^2^	*P*
Age at first marriage	< 18 > = 18	8(5.2%) 146(94.8%)	7(9.1%) 70(90.9%)	1.3	0.26
Age at first childbirth	< 20 > = 20	10(6.5%) 144(93.5%)	8(10.4%) 69(89.6%)	1.1	0.3
Number of pregnancy	< 5 > = 5	70(45.5%) 84(54.5%)	10(13.0%) 67(87.0%)	24	0.000
History of abortion	Yes No	47(30.5%) 107(69.5%)	33(42.9%) 44(57.1%)	3.45	0.063
Number of birth	< 5 > = 5	89(57.8%) 65(42.2%)	18(23.4%) 59(76.6%)	24.5	0.000
Birth space	< 2 > = 2	18(11.7%) 136(88.3%)	23(29.9%) 54(70.1%)	11.6	0.001
place of birth for first child	Home Institution	54(35.1%) 100(64.9%)	47(61.0%) 30(39.0%)	14	0.000
Place of birth for last child	Home Institution	33(21.4%) 121(78.6%)	42(54.5%) 35(45.5%)	25.7	0.000
Instrumental delivery	Yes No	38(24.7%) 116(75.3%)	31(40.3%) 46(59.7%)	6	0.015
Tear during last birth	Yes No	31(20.1%) 123(79.9%)	28(36.4%) 49(63.6%)	7.113	0.008
history of episiotomy	Yes No	41(26.6%) 113(73.4%)	27(35.1%) 50(64.9%)	1.77	0.185
History of induced labor	Yes No	42(27.3%) 112(72.7%)	43(55.8%) 34(44.2%)	18	0.000
fundal pressure during child birth	Yes No	45(29.2%) 109(70.8%)	57(74.0%) 20(26.0%)	41	0.000
Blunt or sharp physical violence/injury on reproductive organ/ perineum	Yes No	32(20.8%) 122(79.2%)	15(19.5%) 62(80.5%)	0.053	0.82
ANC at least ones in all pregnancy	Yes No	125(81.2%) 29(18.8%)	36(46.8%) 41(53.2%)	28.8	0.000
Birth weight of last child	< 4 kg ≥ 4 kg	98 (63.6%) 56 (36.4%)	49 (63.6%) 28 (36.4%	0.0001	1.232
Duration of labor at last birth	4–18 h > 18 h	98(63.6%) 55(35.7%)	54(70.1%) 23(29.9%)	1.35	0.510
Route of birth	SVD Both C/S	107(69.5%) 30(19.5%) 17(11.0%)	63(77.9%) 3(3.9%) 14(18.2%)	11.2	0.004

### Determinants of pelvic organ prolapse

On bivariate logistic regression age, usual work load, educational label, chronic cough, information about POP, number of pregnancy, number of birth, birth interval, duration of return to usual work after childbirth, history of instrumental childbirth, place of birth for last childbirth, mode of birth, history of induced labor, history of fundal pressure and status of menstrual cycle were associated with pelvic organ prolapse with P value less than 0.2. On multivariate logistic regression, heavy usual work load(AOR = 2.3, CI(1.066–4.951), number of pregnancy ≥ 5(AOR = 3.911, CI(1.108–13.802), birth space of < 2 years(AOR = 2.88, CI(1.146–7.232), history of fundal pressure (AOR = 5.312, CI(2.366–11.927) and history of induced labor (AOR = 4.436, CI(2.07–9.505) were significantly associated with POP with P value < 0.05 and 95% CI after adjusting for potential confounders (Table 4).

**Table 4:** Factors associated with pelvic organ prolapse among gynaecologic women in Hawassa city public hospitals, South Ethiopia, 2020

Variables	Category	Cases n = 77	Controls n = 154	COR(95% CI)	AOR(95% CI)
Age	< 40 years	17(22.1%)	76(49.4%)	1	1
	≥ 40 years	60(77.9%)	78(50.6%)	3.439(1.842–6.42)	1.460(0.491–4.346)
Usual work load	Light loaded	25(32.5%)	99(64.3%)	1	1
	Heavy loaded	52(67.5%)	55(35.7%)	3.744(2.097–6.686	2.297(1.066–4.951)
Chronic cough	Yes	33(42.9%)	41(26.6%)	2.067(1.162–3.676)	0.937(0.425–2.123)
	No	44(57.1%)	113(73.4%)	1	1
Information about POP	Yes	20(26.0%)	72(46.8%)	1	1
	No	57(74.0%)	82(53.2%)	2.502(1.374–4.558)	1.810(0.778–4.213)
Pregnancy	< 5	10(13.0%)	70(45.5%)	1	1
	≥ 5	67(87.0%)	84(54.5%)	5.583(2.674–11.659)	3.911(1.108–13.802)
Parity/delivery	< 5	18(23.4%)	89(57.8%)	1	1
	≥ 5	59(76.6%)	65(42.2%)	4.488(2.421–8.319)	0.635(0.190–2.123)
Birth space	< 2 years	23(29.9%)	18(11.7%)	3.218(1.610–6.433)	2.879(1.146–7.232)
	≥ 2 years	54(70.1%)	136(88.3%)	1	1
Place of birth(last delivery)	Home	42(54.5%)	33(21.4%)	4.400(2.436–7.946	1.887(0.735–4.847)
	Health facility	35(45.5%)	121(78.6%)	1	1
Instrumental delivery	Yes	31(40.3%)	38(24.7%)	2.057(1.147–3.691)	1.437(0.622–3.317)
	No	46(59.7%)	116(75.3%)	1	1
Induced labor	Yes	43(55.8%)	42(27.3%)	3.373(1.902–5.98)	4.436(2.070–9.505)
	No	34(44.2%)	112(72.7%)	1	1
Fundal pressure	Yes	57(74.0%)	45(29.2%)	6.903(3.727–12.788)	5.312(2.366–11.927)
	No	20(26.0%)	109(70.8%)	1	1
Return to work after delivery	< 42 days	62(805%)	87(56.5%)	3.183(1.666–6.084)	1.568(0.662–3.718)
	≥ 42 days	15(195%)	67(43.5%)	1	1
Status of menopause	Yes	35(45.5%)	110(71.4%)	1	1
	No	42(54.5%)	44(28.6%)	3.00(1.699–5.298)	0.695(0.263–1.834)

**Fig. 3:**
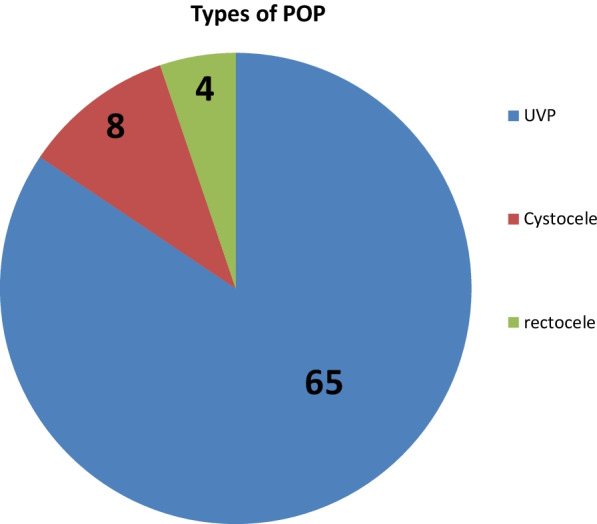
types of pelvic organ prolapse among cases at Public Hospitals in Hawassa City, Southern Ethiopia,

Among cases, 65(84.4%) experienced UVP (Fig. 3). Among those who have UVP, more than one-fourth 22(28.6%) was covered by 3rd degree followed by 4th degree (20(26%) and 2^nd^ degree (19(24.7%). More than half of the cases reported that the duration when they experienced the prolapse was ≤ one year 41(53.2%) and about 36(46.8%) had the duration of > one year.

## Discussion

After adjusting for potential confounders, usual work load, number of pregnancies, duration of birth interval, history of induced labor and fundal pressure during child birth were significantly associated with pelvic organ prolapse.

In this study women’s experiencing heavy usual work (usually heavy load lifting) were 2.3 times more likely to have pelvic organ prolapse than those experiencing light usual work (light load lifting). It was in line presentation to study from Amhara region, Ethiopia by Janne Lille lid Gjerde and colleagues in 2017 which showed that physical strain in women body, during childbirth, work related with food searching or hard physical work can cause serious pelvic wall destruction and then pop and fistula [11]. It was also consistent to study from Wolaita, Tigray and Bahirdar [6, 8, 18]. This might be due to similarity of study population and residence that majority of cases also in my study were from rural residence. It is also consistent to finding of study from Nepal and Tanzania [13, 19, 20]

Women’s with number of pregnancies ≥ 5 were 3.9 times more likely to develop POP than their counterparts in this study. This is congruent to other studies from Ethiopia, Ghana, and Nepal reported that multiple pregnancy/gravidity is a predictor of POP [21, 22]. This is also in line with the fact that repeated pregnancy and birth damages sphincter muscles and ligaments, which sometimes never fully regain its strength and elasticity. Study from Bahirdar shows absence of significant association between gravidity and POP in contrary to this study and it may be due to difference in study participants, and or sample size [8].

Finding of this study also depicts that those women with the birth space of < 2 years were 2.9 times more in high risk of occurrence of POP compared to those with birth space of ≥ 2 years. This supports the scientific hypothesis that frequent vaginal delivery with short birth interval results in significant pelvic floor tissue stretching and pudendal nerve damage in most women which in turn may lead to laxity of pelvic ligaments [3]. The finding is consistently in line with two studies from Nepal [21, 22] but one study from Nepal on reproductive risk factor reported in contrary to this finding that birth spacing had no association with POP. This might be due to difference in study participants or sample size [23]

It was seen in the present study that in comparison with women having no history of induced labor, those with history of induced labor were 4.4 times more likely to experience POP. This was consistent with study from Nepal and was also statistically in line with scientific hypothesis that medical induction of labor might result in highly strong uterine contraction which in turn leads to more painful labor resulting in pressure imbalance on uterus supportive tissue and this pressure differential has important implications for the stresses placed on the support system and cause the tissues to never fully regain its strength and elasticity [3, 24]

In this study, the odds of having POP was 5.3 times more likely among woman’s with history of fundal pressure during their child birth by birth attendants than the counterparts. This is similar to finding from study conducted in Nepal [24]. It was also in line with scientific view that giving pressure or unwanted pulling of baby and pushing of uterus during child delivery might result in perineal tear and great pressure difference on the support system of reproductive organ leading to loss of their strength and elasticity [3].

In addition to the existing study, the finding of this study has great public health importance in the journey of tackling the burden of POP on the life of women since the insight from the finding helps responsible bodies, researchers and obstetric care givers to give due emphasis on significant variables during their clinical practice and research.

### Strength and limitation of the study

This study was one of a few study’s conducted in the country on this topic and using direct primary data by interviewing the respondents to address all the variable of interests including some factors not included in other similar study in the country such as history of diabetes mellitus, physical violence on reproductive organ, fundal pressure during birth and induction of labor were the strength and recall bias for some obstetric characteristic due to long time of duration and being facility based study rather than community based were the limitations.

## Conclusion

Heavy usual work load, having p greater than five pregnancy, short birth space, history of induced labor, and history of fundal pressure are independent predictors of pelvic organ prolapse. Hence the responsible body and obstetric care providers should counsel the women about child spacing, minimizing heavy usual work load and effect of multigravidity on POP. Incorporation of health education on those risk factors related POP on antenatal and postnatal care should be considered. The obstetric care providers also avoid fundal pressure, labor induction without clear indication and favorability and the hospital officials set a law to ban fundal pressure during labor. Further searching with large sample size on risk factors to POP was recommended.

## Data Availability

The data sets that used in this study for analysis and other information are available currently in the hands of the corresponding author and principal investigator. Therefore, it is possible to get with reasonable request**.**

## Abbreviations

POP: Pelvic organ prolapse
AOR: Adjusted odds ratio
COR: Crude odds ratio
CI: Confidence interval
UVP: Utero-vaginal prolapse
SPSS: Statistical package for social science
